# The potential of task shifting selected maternal interventions to auxiliary midwives in Myanmar: a mixed-method study

**DOI:** 10.1186/s12889-017-5020-2

**Published:** 2018-01-03

**Authors:** Kyu Kyu Than, Khaing Nwe Tin, Thazin La, Kyaw Soe Thant, Theingi Myint, James G. Beeson, Stanley Luchters, Alison Morgan

**Affiliations:** 1 Burnet Institute, 85 Commercial Road, Melbourne, VIC 3004 Australia; 2 Department of Medicine, University of Melbourne, Melbourne, Australia; 3 Department of Public Health, Ministry of Health and Sports, Nay Pyi Daw, Myanmar; 4 Department of Epidemiology and Preventive Medicine and Central Clinical School, Monash University, Melbourne, Australia; 5 International Centre for Reproductive Health, Ghent University, Ghent, Belgium; 6 Nossal Institute for Global Health, Melbourne School of Population and Global Health, University of Melbourne, Melbourne, Australia

**Keywords:** Auxiliary midwives, Maternal health, Task shifting, Mixed methods study, Myanmar

## Abstract

**Background:**

An estimated 282 women die for every 100,000 live births in Myanmar, most due to preventable causes. Auxiliary Midwives (AMWs) in Myanmar are responsible for providing a package of care during pregnancy and childbirth to women in rural hard to reach areas where skilled birth attendants (Midwives) are not accessible. This study aims to examine the role of AMWs in Myanmar and to assess the current practices of three proposed essential maternal interventions (oral supplement distribution to pregnant women; administration of misoprostol to prevent postpartum haemorrhage; management of puerperal sepsis with oral antibiotics) in order to facilitate a formal integration of these tasks to AMWs in Myanmar.

**Methods:**

A mixed methods study was conducted in Magwe Region, Myanmar involving a survey of 262 AMWs, complemented by 15 focus group discussions with midwives (MWs), AMWs, mothers and community members, and 10 key informant interviews with health care providers at different levels within the health care system.

**Results:**

According to current government policy, AMWs are responsible for identifying pregnant women, screening for danger signs and facilitating early referral, provision of counselling on nutrition and birth preparedness for women in hard-to-reach areas. AMWs also assist at normal deliveries and help MWs provide immunization services. In practice, they also provide oral supplements to pregnant women (84%), provide antibiotics to mothers during the puerperium (43%), and provide misoprostol to prevent postpartum haemorrhage (41%). The current practices of AMWs demonstrate the potential for task shifting on selected essential maternal interventions. However, to integrate these interventions into formal practice they must be complemented with appropriate training, clear guidelines on drug use, systematic recording and reporting, supportive monitoring and supervision and a clear political commitment towards task shifting.

**Conclusion:**

With the current national government’s commitment towards one AMW in one village, this study highlights the potential for shifting specific maternal lifesaving tasks to AMWs.

## Background

Health workforce shortages are a major contributor to maternal and newborn morbidity and mortality. [1] According to the 2014 census, Myanmar’s maternal mortality ratio was 282 per 100,000 live births with wide variation between urban and rural areas [2]. In Myanmar, there are 14 health care providers per 10,000 persons with the necessary midwifery skills (doctors, nurses and midwives) [3]. This is well below the World Health Organization (WHO) recommendation of at least 23 per 10,000 to achieve 80% coverage for skilled health care worker attendance during deliveries [3, 4].

To address the gap in human resources for health, task shifting has been identified as a promising strategy by WHO to optimize health worker roles to improve access to key maternal and newborn interventions. Task shifting “is a process whereby specific tasks are moved, where appropriate, to health workers with shorter training and fewer qualifications” [1, 5]. Globally, auxiliary health workers such as Myanmar’s Auxiliary Midwives (AMWs) are increasingly becoming providers of health services in low and middle income countries and the services they provide have reduced childhood undernutrition, expanded access to family-planning services and improved maternal and child health [6, 7].

In Myanmar, midwives (MWs) are the primary level health workers within the government health system providing maternal and child health care at the community level. However, MWs are not able to cover all rural villages, where 70% of the population resides [8]. To support MWs to care for mothers and children in hard to reach areas, AMWs are trained and organized by the government [9, 10]. AMWs are unpaid voluntary health workers and have been the largest community level frontline workers serving the mothers and children in rural remote villages since the program commenced in 1978 [11].

The Myanmar Ministry of Health and Sports is in the process of developing an essential package of health services for townships (basic health care infrastructure) that will be covered under universal health coverage. With the commitment of the Myanmar’s government to investing in rural development and the health workforce skill mix, a policy was developed in 2013 of having an AMW in every village of Myanmar [3, 9]. Nationally, there are currently over 30,000 AMWs serving the country, which has a population of around 51.4 million [9, 12].

According to the WHO task-shifting recommendations, the competencies of auxiliary nurse midwife/auxiliary midwife should encompass 10 tasks as outlined in Table 1. Although the definition which includes secondary level of education and a period of on-the-job training for AMWs is similar to WHO’s, AMWs in Myanmar are allowed to conduct only three of the identified tasks. Tasks which involve injection practices are proscribed for AMWs by Ministry of Health and Sports [13]. Within the allowable competencies, three maternal interventions [oral supplementation to pregnant women, oral antibiotics to treat puerperal sepsis, providing misoprostol for prevention of postpartum haemorrhage (PPH)]

**Table 1:** Comparison of the competencies of Auxiliary Midwives in Myanmar with the assumed competencies of Auxiliary Nurse Midwives according to WHO recommendation

Assumed competencies of Auxiliary Nurse Midwives (ANM) within WHO recommendations	Current Auxiliary Midwives competency in Myanmar	Potential competency for Auxiliary Midwives
Promotion of maternal, newborn and reproductive health interventions	**√**	**√**
Oxytocin administration to prevent and treat PPH – standard syringe/ CPAD	Χ	Not allowed due to strict restriction on injection
Misoprostol administration to prevent PPH	Χ	**Possible**
Misoprostol administration to treat PPH	Χ	After the preventions dose must refer the patient immediately to hospital
Oral supplement distribution to pregnant women	Χ	**Possible**
Low dose aspirin distribution to pregnant women at high risk of pre-eclampsia/eclampsia	Χ	After detection of high blood pressure must referred pregnant mothers to the MWs for further care
Continuous support for women during labour, in the presence of a skilled birth attendant	**√**	**√**
Puerperal sepsis management with oral antibiotics	Χ	**Possible**
Puerperal sepsis management with intramuscular antibiotics – CPAD	Χ	Not allowed due to strict restriction on injection
Maternal intrapartum care (including labour monitoring, e.g. using a partograph; foetal heart rate monitoring by auscultation; decision to transfer for poor progress; delivery of the baby)	**√**	**√**

*ANM Auxiliary Nurse Midwives, *PPH* Postpartum haemorrhage, *CPAD* compact pre-filled auto-disable device, *WHO* World Health Organization [Table adapted from the GREAT guideline implementation report for Myanmar, 2014]

were identified for future task shifting by stakeholders (health administrators, policymakers, Non-Government Organizations, professional associations, frontline healthcare providers and researchers) involved in the GREAT (Guideline-driven, Research priorities, Evidence synthesis, Application of evidence, and Transfer of knowledge) network research activity which was carried out in 2014 [14].

However, prior to adding selected tasks to the current work role of AMWs it is important to investigate the current context and understand the practices and perception of the community level actors. The objective of this study is to examine the role of AMWs in Myanmar and to assess the current practices of the three proposed interventions which are not allowed in written policy for the AMWs. We focus on a hard-to-reach area and assess the feasibility of formally integrating these three tasks within AMWs work practices to optimizing their role for maternal health care.

## Methods

### Study setting

Myanmar has critical resource constraints and faces a major gap in access to, and coverage of, health services in many regions of the country. Magwe region in central Myanmar with a population of 3.9 million was purposively selected based on its highest reported national maternal mortality ratio (344 per 100,000 live births) [2]. The region contains a number of hard-to-reach populations and in rural remote areas; most births take place at home. Three townships in Magwe region (Gangaw, Ngape and Seitphyu) were selected for the quantitative survey to represent the geographical diversity of the region and Ngape township was chosen for the qualitative study as the township has a high rate of births with AMWs compared to other townships in the region.

### Study methodology

A mixed methods study design was applied and two previous publications have also reported on the qualitative and quantitative methodology separately [15, 16]. For the qualitative data collection, focus group discussions (FGDs) and key informant interviews (KIIs) were conducted using an interview guide to explore the role of AMWs, attitudes and perception towards AMWs, expectation of the services provided by AMWs and task shifting possibilities. A review of the current manual and micro plan (national plan with detail calculation of how many AMWs would be needed in each state and division, how much cost is needed to train each AMW) for AMWs was conducted. Subsequently, a cross sectional survey of AMWs was carried out using an interviewer administered pre-tested questionnaire, which included the practice of antenatal, birth, postnatal and newborn care practices of AMWs. Results relating to determinants of knowledge of critical danger signs and practices around the time of child birth have been reported in a separate article in which practice of Misoprostol has been mentioned in brief already [16].

### Sampling and recruitment

Purposive sampling was used to collect the qualitative data. A total of ten key informants (three national, two district and three township level health planners and implementers, and two from the Three Millennium Development Goal Fund (3MDG) who were involved in maternal and child health program implementation) were interviewed. Fifteen FGDs (two with MWs, five with AMWs, four with community members and four with mothers with children under the age of 3 years) were conducted.

For the quantitative survey, a list of practicing AMWs was obtained from the township health departments and checked through discussions with the township medical officers. All practicing AMWs in the three townships were contacted for interviews.

All the interviews were conducted in Myanmar language after obtaining written informed consent including an explanation of the study objectives and the intended use of information. Participation was voluntary. Interviews were conducted at a convenient location and the participants were reimbursed with the actual cost of travel to the place of interview and provided a daily allowance to cover meal costs (3000 kyats, equivalent to US$2.5). Confidentiality was maintained throughout the study. Four research assistants led by an experienced researcher conducted the interviews after a three-day training of the research methods and the study objectives. Data collection was done from July 2015 to June 2016.

### Data management and analysis

Quantitative data were checked for consistency and double data entry was done using the software Epi Data version 3.1. Data were analysed using STATA version 13.1. Binary and categorical variables were summarized by proportions and tabulations. For the qualitative data, all the transcripts were read and reread by the principle author in Myanmar language and all the translated versions of the transcripts were read and reread by the other coders. Transcripts of FGDs were translated into English before coding, while remaining data were coded in Myanmar language using ATLAS ti software. Two data coders coded the transcripts. Reliability coding was set at 80% agreement and the inter-coder reliability was found to be over 80%. The analysis was both inductive and deductive and relevant themes were categorized under the three main themes: perception of the role of AMWs, potential for task shifting responsibility and feasibility of integrating new interventions into the current health system. Quotations were used to support the study findings and to enhance understanding of the local context. Data integration and triangulation was done at the interpretation phase of the study using both quantitative and qualitative data.

## Results

### Background characteristics of respondents

A total of 123 people participated in ten key informant interviews and 15 focus group discussions, comprising 15 MWs, 33 AMWs, 29 mothers and 36 community members. The FGDs comprised of 44 (35 women and 9 men) from the hard to reach villages and 69 (61 women and 8 men) from the non-hard to reach villages.

A total of 262 AMWs participated in the quantitative questionnaire survey out of 308 invited to participate (85%). Reasons for non-participation included having a young child at home and poor weather preventing travel. In the very hard to reach villages, 77% of the invited AMWs participated. The majority (82%) of AMWs lived in the villages where they were born (native village). The mean age of AMWs was 32 years and mean duration of working as an AMW was 10 years.

### AMWs current role and responsibility

A review of the current AMWs manual [13] showed that AMWs were mainly responsible to identify pregnant women and make sure that the women are connected with the MWs for four antenatal check-ups including two tetanus toxoid injections. Their main responsibility during pregnancy is to counsel women on healthy eating, danger signs during pregnancy birth preparedness counselling, and early initiation of breast feeding. They are allowed to conduct normal home deliveries, postnatal care and newborn care. They are expected to detect danger signs and promote early referral of women to the MWs, giving first aid care before referral. AMWs conduct health education sessions to the community on nutrition and other locally endemic diseases. Currently they are not allowed to prescribe any medication to pregnant women. They are also responsible for recording and reporting of pregnant women and other cases of disease to the local health authorities (see Table 2).

**Table 2:** Role and responsibilities of Auxiliary Midwife

• Expected to identify pregnant mothers as early as possible and give antenatal care within their agreed authority, ideally aiming for at least four antenatal consultations. (AMW need to refer all registered pregnant women routinely as well as when showing danger signs and necessary must refer a pregnant mother (between 20 weeks and 35 weeks gestation) to the rural health centre for necessary investigation and if needed must accompanied the pregnant mother for emergency referral to the hospital)
• AMW should provide health education to pregnant and lactating women to promote healthy eating and prevention of locally endemic diseases to the community in the village.
• To encourage all pregnant mother to prepare thoroughly for delivery with a comprehensive birth plan
• AMW conducts home deliveries, postnatal care and new born care. Must be able to refer high risk cases of mothers and the newborns defined in the AMW manual to the hospital in timely manner
• AMW should provide support to infants through education to mothers on breast feeding practices such as exclusive breast feeding (for 6 months) and start of supplementary feeding at the age of 6 months.
• Monitor the growth and nutritional status of infants and under five children on a regular basis
• Must provide first aid care in the capacity of her skill and must be able to refer needed cases to the hospital
• Must report unusual diseases to the authority and must record the cases
• Must help and provide assistance to Basic Health Staff in carrying out reproductive health activities.

*AMW = Auxiliary Midwife; [Translated from a Burmese version of Micro plan for auxiliary midwives (2013-2016): Department of Health - Ministry of Health Myanmar, 2013]

### Perception of the role of AMWs

The perception of AMWs by the community and health care providers was positive in the study township and they were considered as the main mediators between the community and health care system. All levels of key informants stated that AMWs are essential health care providers serving the community with not only maternal and child health care but also with other health related activities including disease control and environmental sanitation. Health care providers also mentioned that without the existence of AMWs in hard-to-reach areas where MWs are unavailable, the mothers and children would be in the hands of traditional birth attendants and quacks. MWs in the focus group discussions stated that AMWs were a real helping hand for them because they were able to identify pregnant women in the villages and were able to understand the social background and local language of the women in their care.

*“If they (AMW) do not exist, patients do not have anyone to rely on especially in the hilly villages. You see when a mother gets in trouble during her pregnancy and if AMWs do not exist and we are out of town, there will be lots of problems”* (MW FGD hard-to-reach)

Mothers in the FGDs mentioned that AMWs were the first health care provider they notified of their pregnancy as she is always available. Regular home visits and counselling by AMWs also enhanced the early identification of at risk pregnancies and timely referral. AMWs were also considered as community mobilizers for immunization who make an inclusive list of who is to be immunized. In the hard to reach areas, MWs rely on the AMWs.

*“Well….when they find out new pregnant women, they tell us, I give the pregnant woman antenatal care. They can tell us immediately because they are always around in the village regularly, so, they can get information easily and quickly. They help us when we do health talk sessions and measure babies’ weight. Moreover, they call anyone who needs to receive service and they make sure no one misses.”* (MW FGD non hard-to-reach)

AMWs also conduct normal deliveries in villages where MWs are not present. They are also willing to assist MWs when they conduct deliveries. Post-natal care and psychological support are usually given by AMWs and appreciated by women and their families. AMWs mentioned that serving the community and assisting women in delivery was satisfying. Although they mentioned their role as important, they also expressed their difficulty and devastation of being a voluntary worker without any incentives and payment.

*“We are a common slave for the villagers and the health staff. We don’t have days and nights and whenever there is an emergency they (villagers and health care providers) remember to call us. Sometime we had to come with our own expanse and eat from out of pocket. No one pays us a penny”* (AMW FGD hard-to-reach)

### Potential for task shifting responsibilities

MWs in the study mentioned that they had shared some of their practices with AMWs. The main activities MWs shared to AMWs were providing pregnant women with vitamin supplementation (ferrous sulphate, folic acid, vitamin B1 and vitamin A); providing misoprostol for prevention of PPH; and giving antibiotics such as metronidazole and amoxicillin to treat fever and puerperal sepsis. However, all these activities were done on an ad hoc basis with no regulatory processes. MWs mentioned that these task shifting activities were done due to conditional circumstances and the needs of the community. Geographical distance and the relationship between the AMWs and the MWs were articulated as reasons for task shifting. AMWs that live geographically far away and have a better relationship with the MWs were more likely to be distributing drugs to pregnant mothers compared to those who lived close and were not in a good relationship.

*“For those AMWs in far mountain villages who listen to us, we teach them how to use and what amount should be given for what kind of drugs. We tell them how much should be given for adult and for children. We thoroughly tell them”* (MW FGD hard-to-reach)

Quantitative findings also showed that AMWs were providing the three proposed interventions in practice: oral supplementation (84%), oral antibiotics to treat puerperal sepsis (43%) and misoprostol to prevent postpartum haemorrhage (41%). It was observed that oral supplementation was the most commonly performed task. This was explained in the qualitative findings by AMWs that oral supplementations were more freely available compared to the antibiotics and misoprostol. Health care providers mentioned that, ferrous sulphate, folic acid and vitamin B1 and A were regularly supplied by the government. AMWs in the FGD described,

*“MW usually asks the number of pregnant women. We provide the number and MW gives the medicine (Ferrous sulphate and folic acid) monthly”*

The use of antibiotics for various purposes such as fever, flu and cough was a common practice by both the AMWs and the MWs. The most widely used antibiotics during the puerperal period were amoxicillin and metronidazole. Further exploration was made regarding the dosage of use, and it was mentioned that AMWs were instructed by the MWs to give an adult a dose of 500 mg 3 times per day for amoxicillin and 200 mg 3 times per day for 3 to 5 days for metronidazole. Some MWs mentioned that they use their mobile phones to instruct AMWs on how to treat. The main sources of the drugs were from the MWs and local drug shops. AMWs in the study mentioned that antibiotics can be freely purchased from the local drug stores without need for prescription. A few AMWs in the FGDs mentioned that,

*“we usually buy the drugs from the drug store in town and sometime MWs share their drugs if they get a lot from the township”* (AMW FGD hard-to-reach)

Township level health care providers said that the use of misoprostol to prevent postpartum haemorrhage was introduced around 2012 in the study townships and many of the MWs in the study articulated that they have instructed and shared their misoprostol to AMWs especially those in hard to reach remote villages. The most common dosage of misoprostol by AMWs was two tablets (totalling 400 micrograms) immediately after the birth of the baby. The detailed description of the perception and use of misoprostol by AMWs have been described in an earlier paper [15].

MWs mentioned that with thorough explanation and guidance, they could assure that AMWs would be able to distribute the drugs to mothers safely. AMWs were also confident and willing to take on the assigned role if it was given with proper training and guidance. The potential feasibility of the task shifting activities from the qualitative interviews is outlined in Table 3.

**Table 3:** Responses from FGDs with mothers, community, AMWs and MWs for specific interventions

Task	AMWs (*N* = 33)	MWs (*N* = 15)	Mothers (*N* = 29)	Community (*N* = 36)
Oral supplementation to pregnant women	Confident; Majority of AMWs already in practice, and drugs are mainly supplied by the respective MW during the immunization sessions	Agreed; No difficulty being mentioned	Agreed; Have been taking drugs given by AMWs during pregnancy and childbirth	Agreed; AMWs have been providing oral medication to villagers for minor illnesses
Misoprostol for prevention of PPH	Confident; Some of the AMWs are distributing 2 tablets misoprostol to mothers with drugs provided by MWs especially in hard to reach villages	Agreed; Refresher training suggested on PPH and drug administration	Agreed; Some mothers have received 2 tablets after the birth of the baby from the AMWs, but could not identify the name of the drug	Agreed; Limited knowledge of the drug and if it is for the benefit of the mother and the baby willing to accept
Oral antibiotics for puerperal sepsis	Confident; Only a few AMWs have used antibiotics for puerperal sepsis as cases are rare	Agreed; Refresher training suggested on puerperal sepsis and use of antibiotics	Agreed; Have received some drugs for fever and cough but were not able to identify the name of the drug	Agreed; AMWs have treated fever and cough cases with paracetamol and amoxicillin. Only some were able to identify the name of antibiotics

*AMWs Auxiliary Midwives, *FGDs* Focus Group Discussions, *MWs* Midwives, *PPH* postpartum haemorrhage

The attitude to task shifting was positive by all key informants interviewed. Although many limited the scope of services to oral drugs as most of the health care providers in the study knew that changing the injection drug policy will not be easy and will take time. A district level key informant stated

*“As long as it is not an injection, I think any form of oral drugs like vitamins and antibiotics will be ok. However, we need to train them[AMWs] on how to use the drugs with clear guidelines. Is life saving and it can be given to them safely.”*

### Feasibility of integrating new interventions into the current health system

#### Guidelines on use and availability of drugs

Key informants and MWs raised concerns regarding the instructions and guidelines for use of drugs by AMWs. They mentioned that there are no official guidelines and instructions on use of drugs by either AMWs or MWs. Since there are no guidelines, local townships practices vary according to the availability of the drugs and informal instructions given by individual projects.

They also mentioned the availability and consistent supply of drugs required for task shifting. Currently, there is ample supply of drugs due to the government supply in 2015 and the 3MDG program, however MWs stated that regular and consistent supply of drugs will be needed for sustainability and effective task shifting. For the AMWs, the supply of drugs [such as ferrous sulphate, folic acid, B1, misoprostol and antibiotics (amoxicillin and metronidazole)] was currently given by the MWs but the supply is inconsistent. There is neither systematic supply chain mechanism for the AMWs nor any recording of drugs given which was articulated as a barrier to potential task shifting.

*“We (MWs) just give the drugs when we have it and when we don’t we cannot give it, they (AMWs) also get the drugs direct from the NGO working in the area, sometime”* (MW FGD non hard-to-reach)

#### Inconsistent MW availability

Key informants mentioned that task shifting of the proposed interventions was feasible in places where there is inconsistent availability of MWs. Although MWs are assigned to hard to reach villages where there is a sub rural health centre, they rarely live in the assigned villages due to transportation and socioeconomic difficulties. This inconsistent MW availability was also articulated by the mothers and community members in the FGDs. In one hard to reach village the MW had been absent throughout her assigned period. MWs also expressed their difficulties of staying in hard to reach villages due to challenging transportation, language barriers, poor living conditions and cost of living.

*“If you are not a native, the very first problem is language. We can’t live because their life styles are not the same….quite frankly, one who is not local will only stay for 15 days in an assigned area while local stays because the local one doesn’t need to return home”* (MW FGD hard-to-reach)

#### Supervision and monitoring issues

All levels of health care providers pointed out that current supervision and monitoring systems for AMWs were weak. Apart from MWs meeting with AMWs during the monthly immunization sessions, there was no regular planned supervision or monitoring visits. Lack of support for travel was identified as the major barrier by MWs for supervision. Some of the AMWs mentioned that no government health staff had ever visited their villages. Many of the MWs mentioned that although they realize the importance of supervision and monitoring, there is no support mechanism currently available to solve the problem.

*“No one from the health department has ever been to my village because it is very far and it takes me about 9 hours to get here but for you it may take the whole day”* (AMW FGD hard-to-reach)

#### Training gaps

Many key informants and AMWs in the study talked about the need for proper training if the new practices were to be implemented as there is no standard guideline on how to use the drug and dosage in the current training manual. Key informants also raised concerns regarding the current training materials and methods of training stating they were too theoretical and that the current AMWs’ manual resembles a text book rather than a training curriculum. There is no Training of trainers for AMWs at the national level. In regard to training for potential task shifting interventions, respondents emphasised practical training with hands on exercises.

*“For the AMW, we must have a specific curriculum for the training and all the oral drugs usage needs to be in the guideline, it should be standardized from the Department of Health Human Resource and Planning. We need one trainer and trainee manual for the whole country. It should not be project or funding based. It is the responsibility of the health system to control the trainings”* (District level key informant)

#### Health system priorities

Maternal and child health has been given a high priority by the government in recent years. Key informants from the national and district level mentioned that task shifting is a new word for them and that there are policy level limitations and delays that may be faced in introducing task shifting to AMWs. They suggested that the AMW program reached its peak in recent years due to political interest by the previous government. However, with the changing political landscape after the 2016 election, it is not clear if this support will continue. They stated that evidence based policy briefs will be necessary for the potential task shifting interventions to move forward with increasing awareness and interest. A national level key informant said that,

*“At the moment AMW trainings are continuing but I think is not a priority anymore after the change of government and there is no clear policy….MW upgrading and curriculum development…. new Public Health Supervisor 2 trainings…is all here and there*”

## Discussion

The findings from this study highlight that AMWs in Myanmar are already demonstrating their capacity to provide selected maternal health interventions. Despite the absence of policy endorsement there is a local acceptance by MWs who facilitate AMWs to provide drugs to mothers in hard to reach areas. The current role of AMWs, as intermediaries between the community and the health system and their accepted place in antenatal care, childbirth and postnatal care translates to broad acceptability for giving oral medications to women during pregnancy and at the time of child birth.

Another factor in favour of task shifting is the inconsistent availability of the MWs in geographically hard to reach villages. Although the Ministry of Health and Sports recognises that more MWs are needed, there are still limitations to filling the gap [3]. Other studies have also shown that skilled health care providers are not available in the assigned health posts due to financial and transport difficulties, socioeconomic hardship, available schooling and geographical location [17]. Generally, AMWs in geographical locations where there are no MWs and no support system were more in favour of task shifting [15, 18]. Task shifting to alternative cadres for maternal and child health has been emphasized as an important strategy in countries where there is geographical inaccessibility and limited availability of skilled care attendants [19–21].

Other country experiences demonstrate that administration of medications by community based health workers has reduced childhood and maternal morbidity and mortality [22–24]. Trained community volunteers in Nepal were able to provide misoprostol to women in the community and uterotonic protection for deliveries rose from 11.6% to 74.2% with largest gain to reach the poor, the illiterate and women from remote areas [25]. A study in Pakistan using Trained Traditional Birth Attendants with monitoring supported by Lady Health Visitors and Community Health Nurses, to administer 600 microgram of misoprostol to women with postpartum haemorrhage has reduced maternal mortality by 24% [26]. However, these studies also recommended the importance of training and supportive supervision as a necessity in success [22, 25, 26].

In our study task shifting was already occurring in the study townships. Although such informal task shifting has been suggested as an initial path to task shifting, informal task shifting without having an explicit policy has raised concerns by health care workers and policy makers regarding unclear role and responsibilities of health workers [27–29]. Without the protection of a formalized policy and guidelines in practice, task shifting may pose a threat to the safety and performance of AMWs.

In other reviews of task shifting programs recommendations include conducting a detailed task analysis of both the work of those to whom tasks will be shifted and the work of those from who tasks will be relieved, to ensure that the task shifting does not create a different system bottlenecks [29, 30]. Although this study reviewed the tasks provided by AMWs, the study was limited to this cadre only without a comprehensive review of tasks performed by MWs. A task analysis of existing community level providers for maternal and child health care is recommended for the effective implementation of the task shifting interventions [30, 31]. Also task analysis provides the opportunity to revisit the current voluntary status as the increasing complexity of tasks expected of AMWs may not be consistent with a voluntary status. Thus incentives and funding mechanism for AMWs need to be considered in future policy options.

Despite task shifting being a practicable option for human resource shortage, reports of concerns regarding quality, safety and sustainability have been documented from experiences of task shifting in HIV/AIDS in sub-Saharan Africa [32, 33]. Lessons learnt from barriers and enablers to effective implementation of the guidelines has pointed out that without evidence-based health policies to support implementation along with health system level factor readiness it will be hard to effectively implement task shifting initiatives [34].

Although there is an AMW manual [13], many of the health care providers in the study mentioned that no training curriculum for both trainers and trainees regarding AMWs existed. Reviews have pointed out that without appropriate training resources (curriculum, trainers and training materials) and appropriate skills building, task shifting may place the low level health worker and their supervisors at risk of malpractice [31]. In eastern Myanmar, task shifting to promote basic health service delivery among internally displaced people in ethnic health program service areas showed success through reorganizing and training the workforce with a rigorous and up-to-date curriculum for the Ethnic health organizations and community based health organizations [35]. According to the new National Health Plan, the government is committed to ongoing capacity building: “Ensuring that all Voluntary Based Health Workers, including CHWs and AMWs, are confident to take on the duties assigned to them is critical to their effectiveness. Sufficient skill-based training and consistent support and supervision from Basic Health Staff will be required – this requires resources to support and it needs to be taken into account for the uptake and delivery of interventions” [36].

### Strengths and limitations of the study

We employed a mixed method approach which enriched the study in two ways. First, the qualitative study helped us to understand the potential for task shifting three interventions to AMWs from both community and providers’ perspectives. The feasibility of the tasks was confirmed by the quantitative study where we documented the frequency with which these tasks were already being performed by AMWs. However, the study townships were purposefully selected based on their hard-to-reach characteristics and findings may not be representative of the perspectives of equivalent stakeholders in other regions, limiting generalizability of our findings.

## Conclusions

Our study brings light to the Myanmar context in considering the potential for training and task shifting the oral administration of essential maternal interventions at community level to AMWs. The necessary enabling factors that are described above include supportive supervision, clear drug usage guidance and procurement and policy commitment to task shifting. As the Ministry of Health and Sports is committed to increasing the number of AMWs in every village of the country for reaching equitable care to mothers and newborn, optimizing the role of AMWs through task shifting effective interventions on maternal and child health should be considered.

## Abbreviations

3MDG: The three millennium development goal fund
AMW: Auxiliary midwife
CPAD: Compact pre-filled auto-disable device
FGD: Focus group discussion
GREAT: Guideline-driven, research priorities, evidence synthesis, application of evidence, and transfer of knowledge
KII: Key informant interview
MW: Midwife
NGO: Non-government organization
PPH: Postpartum haemorrhage
WHO: World Health Organization

## References

[1] World Health Organization: WHO recommendations: Optimizing health worker roles to improve access to key maternal and newborn health interventions through task shifting OPTIMINIZEMNH. In. Geneva: World Health Organization; 2012. 23844452

[2] Department of Population-Ministry of Labour Immigration and Population-The Republic of the Union of Myanmar: Thematic report on Maternal Mortality, Census Report Volume 4-C. In., vol. Census Report Volume 4-C. Nay Pyi Daw: Department of Population-Ministry of Labour Immigration and Population; 2016.

[3] Ministry of Health Myanmar: Health Workforce Strategic Plan 2012-2017. In.; 2012.

[4] World Health Organization: World Health Report (2006). Working together for health.

[5] World Health Organization: Task shifting: rational redistribution of tasks among health workforce teams: global recommendations and guidelines. Geneva: World Health Organization; 2007.

[6] Gilmore B, McAuliffe E (2013). Effectiveness of community health workers delivering preventive interventions for maternal and child health in low- and middle-income countries: a systematic review. BMC Public Health.

[7] Perry HB, Zulliger R, Rogers MM (2014). Community health workers in low-, middle-, and high-income countries: an overview of their history, recent evolution, and current effectiveness. Annu Rev Public Health.

[8] Department of Population-Ministry of Immigration and Population -The Republic of the Union of Myanmar: Country Report on 2007 Fertility and Reproductive Health Survey. In. Yangon: Department of Population; 2009.

[9] Department of Health - Ministry of Health Myanmar: Microplan for auxiliary midwives (2013-2016) (Myanmar Language). In. Nay Pyi Daw: Department of Health; 2013.

[10] Department of Health - Ministry of Health Myanmar: Assessment of performance and acceptablity of Auxiliary Midwives in rural communities as a strategy to improve maternal health In.; 2005.

[11] Department of Health - Ministry of Health Myanmar: Situational analysis on training and utilization of Auxiliary Midwives. In. Yangon: Department of Health; 1985.

[12] Department of Population-Ministry of Labour Immigration and Population-The Republic of the Union of Myanmar: The 2014 Myanmar Population and Housing Census, The Union Report, Census Report Volume 2. In., vol. Census Report Volume 2. Nay Pyi Taw: Department of Population-Ministry of Labour Immigration and Population; 2015.

[13] Department of Health - Ministry of Health Myanmar: Auxiliary Midwife Training Manual (Myanmar Language). In. Nay Pyi Daw: Department of Health; 2015.

[14] Khan S, Timmings C, Vogel J, Thike KB, Moore J, Gülmezoglu M, Tran N, Straus S: GREAT Project [Guideline-driven, Research priorities, Evidence synthesis, Application of evidence, and Transfer of knowledge]. 2014.

[15] Than KK, Mohamed Y, Oliver V, Myint T, La T, Beeson JG, Luchters S (2017). Prevention of postpartum haemorrhage by community-based auxiliary midwives in hard-to-reach areas of Myanmar: a qualitative inquiry into acceptability and feasibility of task shifting. BMC Pregnancy and Childbirth.

[16] Than KK, Morgan A, Pham MD, Beeson JG, Luchters S. Determinants of knowledge of critical danger signs, safe childbirth and immediate newborn care practices among auxiliary midwives: a cross sectional survey in Myanmar. BMJ Open. 2017:7(6). 10.1136/bmjopen-2017-017180 PMC5734551 28679678

[17] Oo K, Win L, Saw S, Mon M, Oo Y, Maung T (2012). Challenges faced by skilled birth attendants in providing antenatal and intrapartum care in selected rural areas of Myanmar. WHO South-East Asia J Public Health.

[18] Wangmo S, Suphanchaimat R, Htun WMM, Tun Aung T, Khitdee C, Patcharanarumol W, Htoon PT, Tangcharoensathien V (2016). Auxiliary midwives in hard to reach rural areas of Myanmar: filling MCH gaps. BMC Public Health.

[19] Glenton C, Colvin CJ, Carlsen B, Swartz A, Lewin S, Noyes J, Rashidian A. Barriers and facilitators to the implementation of lay health worker programmes to improve access to maternal and child health: qualitative evidence synthesis. Cochrane Database Syst Rev. 2013;10 10.1002/14651858.CD010414.pub2 PMC6396344 24101553

[20] Adam MB, Dillmann M, Chen MK, Mbugua S, Ndung'u J, Mumbi P, Waweru E, Meissner P (2014). Improving maternal and newborn health: effectiveness of a community health worker program in rural Kenya. PLoS One.

[21] Byrne A, Hodge A, Jimenez-Soto E, Morgan A. What works? Strategies to increase reproductive, maternal and child health in difficult to access mountainous locations: a systematic literature review. PLoS ONE [Electronic Resource]. 2014;9(2):e87683. 10.1371/journal.pone.0087683 PMC3912062 24498353

[22] Lassi ZS, Das JK, Salam RA, Bhutta ZA (2014). Evidence from community level inputs to improve quality of care for maternal and newborn health: interventions and findings. Reprod Health.

[23] Dawson AJ, Buchan J, Duffield C, Homer CS, Wijewardena K (2013). Task shifting and sharing in maternal and reproductive health in low-income countries: a narrative synthesis of current evidence. Health Policy Plan.

[24] Haines A, Sanders D, Lehmann U, Rowe AK, Lawn JE, Jan S, Walker DG, Bhutta Z (2007). Achieving child survival goals: potential contribution of community health workers. Lancet.

[25] Rajbhandari S, Hodgins S, Sanghvi H, McPherson R, Pradhan YV, Baqui AH (2010). Expanding uterotonic protection following childbirth through community-based distribution of misoprostol: operations research study in Nepal. Int J Gynecol Obstet.

[26] Mobeen N, Durocher J, Zuberi NF, Jahan N, Blum J, Wasim S, Walraven G, Hatcher J (2011). Administration of misoprostol by trained traditional birth attendants to prevent postpartum haemorrhage in homebirths in Pakistan: a randomised placebo-controlled trial. BJOG Int J Obstet Gynaecol.

[27] Dovlo D (2004). Using mid-level cadres as substitutes for internationally mobile health professionals in Africa. Desk Rev Hum Resour Health.

[28] Nabudere H, Asiimwe D, Mijumbi R (2011). Task shifting in maternal and child health care: an evidence brief for Uganda. Int J Technol Assess Health Care.

[29] Zachariah R, Ford N, Philips M, Lynch S, Massaquoi M, Janssens V, Harries AD (2009). Task shifting in HIV/AIDS: opportunities, challenges and proposed actions for sub-Saharan Africa. Trans R Soc Trop Med Hyg.

[30] Fulton BD, Scheffler RM, Sparkes SP, Auh EY, Vujicic M, Soucat A (2011). Health workforce skill mix and task shifting in low income countries: a review of recent evidence. Hum Resour Health.

[31] Deller B, Tripathi V, Stender S, Otolorin E, Johnson P, Carr C (2015). Task shifting in maternal and newborn health care: key components from policy to implementation. Int J Gynecol Obstet.

[32] Dambisya YM, Matinhure S (2012). Policy and programmatic implications of task shifting in Uganda: a case study. BMC Health Serv Res.

[33] Callaghan M, Ford N, Schneider H (2010). A systematic review of task- shifting for HIV treatment and care in Africa. Hum Resour Health.

[34] Vogel JP, Moore JE, Timmings C, Khan S, Khan DN, Defar A, Hadush A, Minwyelet Terefe M, Teshome L, Ba-Thike K (2016). Barriers, facilitators and priorities for implementation of WHO maternal and Perinatal health guidelines in four lower-income countries: a GREAT network research activity. PLoS One.

[35] Low S, Tun KT, Mhote NPP, Htoo SN, Maung C, Kyaw SW, Shwe Oo SEK, Pocock NS: Human resources for health: task shifting to promote basic health service delivery among internally displaced people in ethnic health program service areas in eastern Burma/Myanmar. Global Health Action 2014, 7: 10.3402/gha.v3407.24937. 10.3402/gha.v7.24937 PMC4185086 25280737

[36] Ministry of Health and Sports-The Republic of the Union of Myanmar: Myanmar national health plan (2017-2020). In. Nay Pyi Daw: Ministry of Health and Sports; 2016.

